# Stoichiometric and polymorphic salts of hexa­methyl­ene­tetra­minium and 2-chloro-4-nitro­benzoate

**DOI:** 10.1107/S2056989017014359

**Published:** 2017-10-13

**Authors:** Andreas Lemmerer, Xolani Motlaung

**Affiliations:** a Molecular Sciences Institute, School of Chemistry, University of the Witwatersrand, Private Bag, PO WITS, 2050, Johannesburg, South Africa

**Keywords:** crystal structure, mol­ecular salts, stoichiometric ratio, polymorphs

## Abstract

Four mol­ecular salts made from hexa­methyl­ene­tetra­minium and 2-chloro-4-nitro­benzoate have been synthesized and are reported, namely ammonium hexa­methyl­ene­tetra­minium bis­(2-chloro-4-nitro­benzoate), NH_4_^+^·C_6_H_13_N_4_^+^·2C_7_H_3_ClNO_4_^−^, (I), hexa­methyl­ene­tetra­minium hydrogen bis­(2-chloro-4-nitro­benzoate), 0.5C_6_H_13_N_4_^+^·C_7_H_3.50_ClNO_4_^−^, (II), hexa­methyl­ene­tetra­minium 2-chloro-4-nitro­benzoate, C_6_H_13_N_4_^+^·C_7_H_3_ClNO_4_^−^, (III*a*) and (III*b*). All four mol­ecular salts show N^+^—H⋯O^−^ hydrogen bonding. Salt (I) crystallized out with an NH_4_^+^ counter-ion which came from decomposition of 50% of the hexa­methyl­ene­tetra­minium cation in solution. (II) shows an unusual asymmetric unit, with both a hexa­methyl­ene­tetra­minium cation and a partially deproton­ated 2-chloro-4-nitro­benzoate anion. Salts (III*a*) and (III*b*) are polymorphs of each other. This work shows that hexamethylenetetramine only protonates once, even in the presence of excess acid.

## Abstract 2

Four mol­ecular salts made from hexa­methyl­ene­tetra­minium and 2-chloro-4-nitro­benzoate have been synthesized and are reported. All four mol­ecular salts show N^+^—H⋯O^−^ hydrogen bonding. This work shows that hmta only protonates once, even in the presence of excess acid.

## Chemical context   

Crystal engineering, the conception and synthesis of mol­ecular solid-state structures, is fundamentally based upon the discernment and subsequent exploitation of inter­molecular inter­actions (Desiraju, 1989 ▸) Thus, primarily non-covalent bonding is used to achieve the organization of mol­ecules and ions in the solid state in order to produce materials with desired properties. One mol­ecule that has been used that has multiple acceptor sites is hexa­methyl­ene­tetra­mine (hmta), and it has been shown to act as a hydrogen-bond acceptor for alcohol or carb­oxy­lic acid donors (Lemmerer, 2011 ▸). Inter­estingly, hmta has four equivalent N atoms but there are very few reported co-crystals or salts that use all four. Examples that use all four N atoms in neutral hydrogen bonding are seen with alcohols (MacLean *et al.*, 1999 ▸), whereas the vast majority of mol­ecular complexes with hmta show it acting as a twofold acceptor (Li *et al.*, 2001 ▸). However, if protonation does occur, then it is usually confined to only one site being protonated (Lemmerer *et al.*, 2012 ▸). 2-Chloro-4-nitro­benzoic (2c4nH) acid has been used extensively in making co-crystals and salts using pyridine as an acceptor (Lemmerer *et al.*, 2010 ▸, 2015 ▸) and has been chosen to be the hydrogen-bond donor/acid. The experimental p*K*_a_ of hmta is 4.89 (Cooney *et al.*, 1986 ▸), and the calculated p*K*_a_ of 2c4nH is 2.04 (Lemmerer *et al.*, 2015 ▸). Childs *et al.* (2007 ▸) postulated that for 0 < Δp*K*_a_ < 3, either a neutral co-crystal or salt can form, and that the crystalline environment can influence which one is favoured. In general, however, for Δp*K*_a_ values > 3 and < 0, a salt or co-crystal, respectively, is formed (Lemmerer *et al.*, 2015 ▸). Hence, it is postulated that proton transfer will occur for a solution containing hmta and 2c4nH. In this work, we will make mol­ecular salts using a 1:1 or 1:2 ratio of hmta with 2c4nH to see if two N atoms sites can be protonated. The four salts synthesized and reported here are: (hmtaH^+^)·(NH_4_^+^)(2c4nH^−^)_2_, (I), (hmtaH^+^)·(2c4nH^−^)_2_, (II) and (hmtaH^+^)·(2c4nH^−^), (III*a*) and (III*b*).
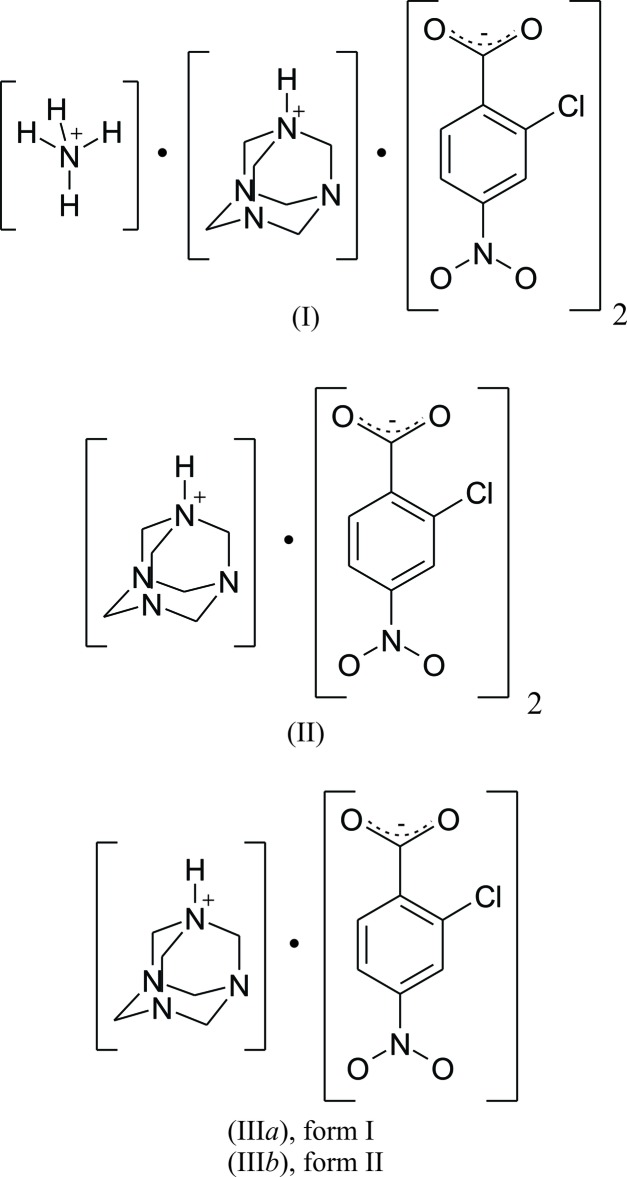


## Structural commentary   

**Figure 1:**
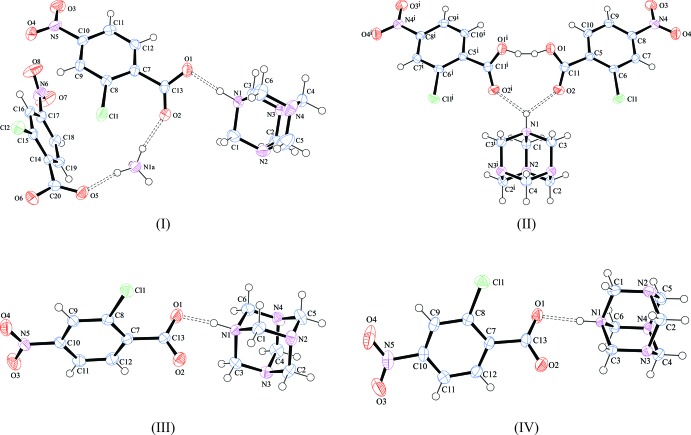
Perspective views of compounds (I)–(III*b*), showing the atom-numbering schemes. Displacement ellipsoids are drawn at the 50% probability level and H atoms are shown as small spheres of arbitrary radii. Atoms with superscript (i) are at symmetry position (*x*, −*y* + 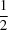, *z*). The dashed lines indicate the symmetry-independent N^+^—H⋯O^−^ hydrogen bonds.

The asymmetric units and atom-labelling schemes are shown in Fig. 1 ▸, together with their displacement ellipsoids for all four salts. A noteworthy asymmetric unit is the one for salts (I) and (II). In salt (I), there is the expected simple hmtaH^+^ cation and 2c4n^−^ pair that are hydrogen bonded to each other using a charge-assisted N^+^—H⋯O^−^ hydrogen bond (Table 1 ▸). However, an NH_4_^+^ ammonium cation is included in the asymmetric unit and its charge is balanced by a second 2c4n^−^ anion. The NH_4_^+^ cation’s appearance is not unique as it has been reported in the literature that hmta can decompose to form NH_4_ and formaldehyde (Lough *et al.*, 2000 ▸), especially if the crystallization takes place slowly and in the presence of an acid. From a crystallographic standpoint, the 2:1 mol­ecular salt (II) features half of an hmtaH^+^ cation crystallizing along a mirror plane at *y* = 1/4 and a fully occupied 2c4nH anion. In the difference-Fourier map, there is clear evidence that the N1 atom on a special position (0.485286 0.250000 0.494001) is protonated and hence has a half positive charge. However, the carb­oxy­lic acid group of 2c4nH has bond lengths typical of being neutral and clearly shows an acidic H atom, H2, located near O1 in the difference-Fourier map. Combined, this means that H1 acts as a bifurcated donor to two 2-chloro-4-nitro­benzoic mol­ecules (Table 2 ▸), which themselves share the hydrogen atom H2. Mol­ecular salts (III*a*) and (III*b*) both have a 1:1 ratio and are polymorphs of each other. Both have charge-assisted N^+^—H⋯O^−^ hydrogen bonds (Tables 3 ▸ and 4 ▸) between the two ions but differ in their packing as described further below.

## Supra­molecular features   

**Figure 2:**
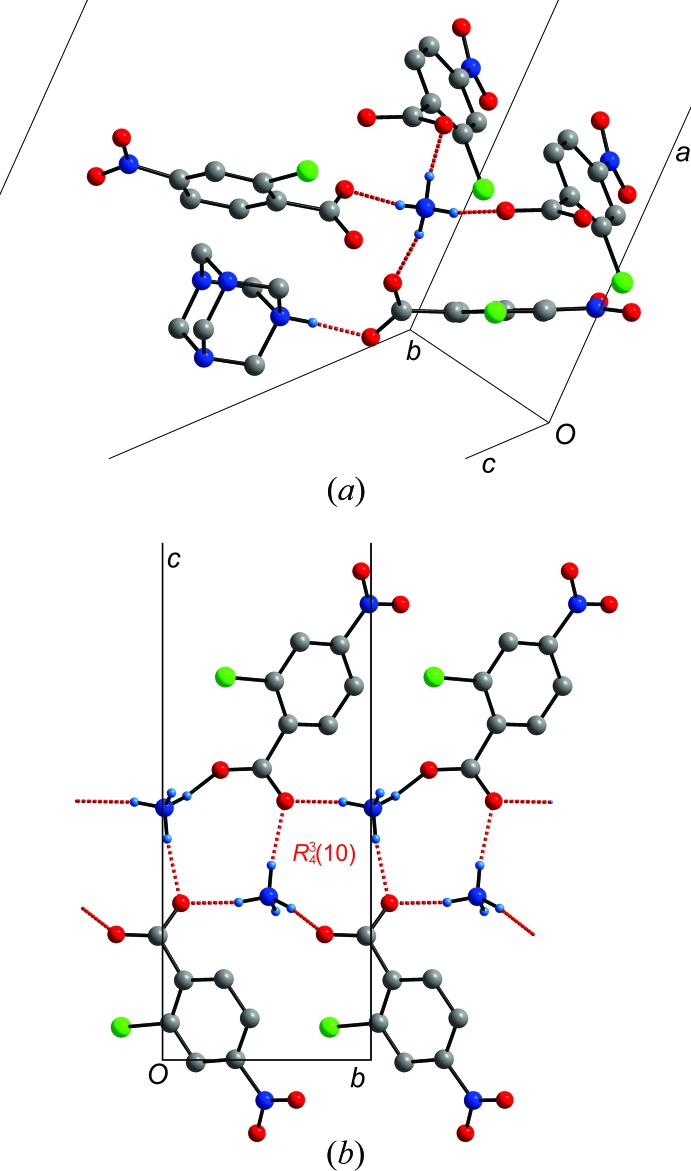
(*a*) Detailed view of the five hydrogen bonds formed by the cations and anions in (I). (*b*) The hydrogen-bonded ladder formed between the NH_4_^+^ cation and carboxyl­ate anion forming a repeating 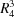
(8) motif. Hydrogen bonds are shown as dashed red lines.

**Figure 3:**
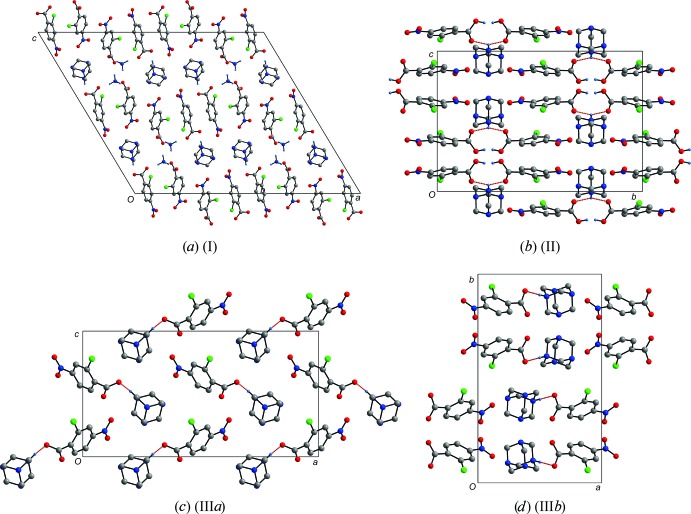
The packing diagrams for all four salts. Note the different packing arrangement of the two 1:1 dimorphs (III*a*) and (III*b*).

The packing of salt (I) consists of clearly separated layers of hydro­phobic and hydro­phillic layers. All good hydrogen-bond donors are used (Table1, Fig. 2 ▸ *a*). The NH_4_^+^ cation forms a hydrogen-bonded ring using two carboxyl­ate groups and this ring repeats along the *b*-axis direction. The ring can be described as 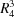
(8) and is a common feature in ammonium carboxyl­ate salts (Lemmerer & Fernandes, 2012 ▸). This ladder is then surrounded by a 2c4n^−^ anion that hydrogen bonds to the hmta^+^ cation. Overall, the hydro­philic layer consists of the cationic NH part of hmtaH^+^, NH_4_^+^ and the carboxyl­ate CO_2_^−^ part of 2c4n^−^ (Fig. 3 ▸ *a*). Salt (II) consists only of the hmtaH^+^ and 2c4n^−^ anion in a 1:2 ratio. However, it appears crystallographically that only one complete proton transfer has taken place, and that on average, each of the 2c4n anions has released half a proton each to the N atom (labelled H1) and that the other half proton (labelled as H2) is located in between the two anions. Hence, only one N atom on hmta has been protonated, and subsequently, two 2c4n^−^ anions are behaving as acceptors from a single N—H group (Fig. 1 ▸). Overall, the same layering of hydro­philic and hydro­phobic parts occurs, where the cationic and anionic parts are located in the same *ac* plane. Salts (III*a*) and (III*b*) have identical asymmetric units with a 2:1 ratio of hmtaH^+^ and 2c4n^−^, in contrast to the previous two salts. The only significant difference is in the relative packing of these ion pairs. In (III*a*), the pairs pack anti-parallel (Fig. 3 ▸ *c*), and in (III*b*), parallel (Fig. 3 ▸ *d*).

## Database survey   

Up to now, there are only 36 structures of singly protonated hmtaH^+^ mol­ecular salts in the Cambridge Structural Database (CSD, Version 5.38; Groom *et al.*, 2016 ▸), together with any organic or inorganic counter-anion. Only one structure has the hmta doubly protonated (FOQZIW; Zaręba *et al.*, 2014 ▸). Co-crystals of hmta in a 1:1 or 1:2 ratio with carb­oxy­lic acids are much more numerous (45). Ultimately, it has been shown that even with an excess of 2c4n, the hmta mol­ecule only allows itself to be protonated once.

## Synthesis and crystallization   

**Figure 4:**
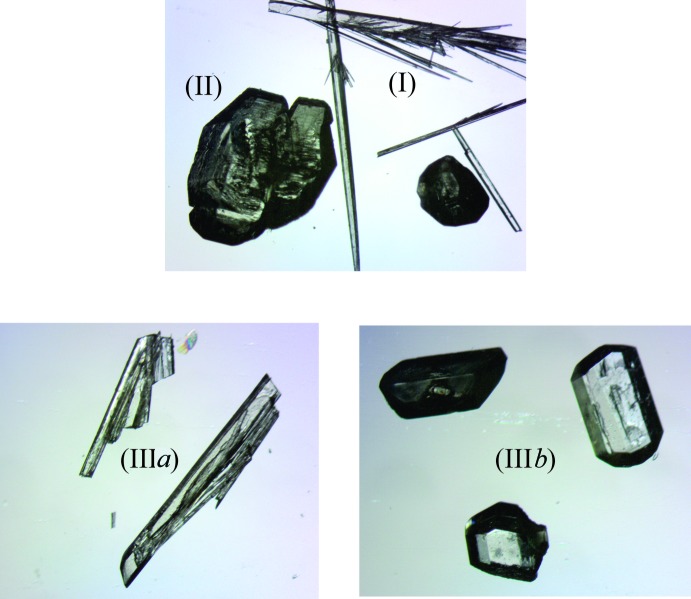
The morphologies of the four title salts: (I) block, (II) plate, (III*a*) thick needles and (III*b*) prism.

All chemicals were purchased from commercial sources (Sigma Aldrich) and used as received without further purification. Crystals were grown *via* the slow evaporation method, under ambient conditions, of alcoholic solutions. For (I) and (II), these crystals crystallized out concomitantly from a 1:2 ratio, and (III*a*) and (III*b*), concomitantly from a 1:1 molar ratio. The morphology of the yellow-tinted crystals are shown in Fig. 4 ▸. Detailed masses and volumes are as follows. For (I) and (II): hexa­methyl­ene­tetra­mine (0.050 g, 0.375 mmol) and 2-chloro-4-nitro­benzoic acid acid (0.072 g, 0.375 mmol) in methanol (5 mL); for (III*a*) and (III*b*): hexa­methyl­ene­tetra­mine (0.050 g, 0.375 mmol) and 2-chloro-4-nitro­benzoic acid acid (0.144 g, 0.750 mmol) in ethanol (5 mL).

## Refinement details   

Crystal data, data collection and structure refinement details are summarized in Table 5 ▸. For all compounds, the C-bound H atoms were placed geometrically (C—H bond lengths of 0.99 (ethyl­ene CH_2_), and 0.95 (Ar—H) Å) and refined as riding with *U*_iso_(H) = 1.2*U*_eq_(C). The N–bound H atoms were located in difference-Fourier maps and their coordinates and isotropic displacement parameters allowed to refine freely. The O–bound H atom in (II) was located in the difference-Fourier map and refined as riding with *U*_iso_(H) = 1.5*U*_eq_(O).

## Supplementary Material

Crystal structure: contains datablock(s) I, II, IIIa, IIIb, shelx. DOI: 10.1107/S2056989017014359/eb2001sup1.cif

Structure factors: contains datablock(s) I. DOI: 10.1107/S2056989017014359/eb2001Isup2.hkl

Structure factors: contains datablock(s) II. DOI: 10.1107/S2056989017014359/eb2001IIsup3.hkl

Structure factors: contains datablock(s) IIIa. DOI: 10.1107/S2056989017014359/eb2001IIIasup4.hkl

Structure factors: contains datablock(s) IIIb. DOI: 10.1107/S2056989017014359/eb2001IIIbsup5.hkl

CCDC references: 1578099, 1578098, 1578097, 1578096

Additional supporting information:  crystallographic information; 3D view; checkCIF report

## Figures and Tables

**Table 1:** Hydrogen-bond geometry (Å, °) for (I)

*D*—H⋯*A*	*D*—H	H⋯*A*	*D*⋯*A*	*D*—H⋯*A*
N1—H1⋯O1	0.94 (2)	1.71 (2)	2.6564 (18)	177 (2)
N1*A*—H1*A*⋯O2	0.94 (2)	1.89 (2)	2.817 (2)	168 (2)
N1*A*—H2*A*⋯O5	0.93 (2)	1.87 (2)	2.784 (2)	167 (2)
N1*A*—H3*A*⋯O5^i^	0.91 (2)	1.90 (2)	2.803 (2)	171 (2)
N1*A*—H4*A*⋯O6^ii^	1.02 (2)	1.73 (2)	2.747 (2)	173 (2)

Symmetry codes: (i) 
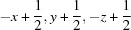
; (ii) 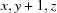.

**Table 2:** Hydrogen-bond geometry (Å, °) for (II)

*D*—H⋯*A*	*D*—H	H⋯*A*	*D*⋯*A*	*D*—H⋯*A*
N1—H1⋯O2	0.92 (3)	2.12 (2)	2.7667 (15)	126 (1)

**Table 3:** Hydrogen-bond geometry (Å, °) for (III*a*)

*D*—H⋯*A*	*D*—H	H⋯*A*	*D*⋯*A*	*D*—H⋯*A*
N1—H1⋯O1	1.00 (3)	1.60 (3)	2.599 (2)	173 (3)

**Table 4:** Hydrogen-bond geometry (Å, °) for (III*b*)

*D*—H⋯*A*	*D*—H	H⋯*A*	*D*⋯*A*	*D*—H⋯*A*
N1—H1⋯O1	0.90 (2)	1.80 (2)	2.6911 (17)	175.7 (19)

**Table 5:** Experimental details

	(I)	(II)	(III*a*)	(III*b*)
Crystal data				
Chemical formula	H_4_N^+^·C_6_H_13_N_4_^+^·2C_7_H_3_ClNO_4_^−^	0.5C_6_H_13_N_4_^+^·C_7_H_3.50_ClNO_4_^−^	C_6_H_13_N_4_^+^·C_7_H_3_ClNO_4_^−^	C_6_H_13_N_4_^+^·C_7_H_3_ClNO_4_^−^
*M*_r_	560.35	543.32	341.76	341.76
Crystal system, space group	Monoclinic, *C*2/*c*	Orthorhombic, *P* *n* *m* *a*	Monoclinic, *C* *c*	Monoclinic, *P*2_1_/*c*
Temperature (K)	173	173	173	173
*a*, *b*, *c* (Å)	33.6032 (8), 6.0235 (1), 28.0229 (7)	8.2777 (2), 19.7942 (5), 13.5331 (4)	5.9049 (1), 21.9330 (4), 12.0194 (2)	12.0663 (2), 19.5741 (4), 6.6473 (1)
α, β, γ (°)	90, 121.007 (1), 90	90, 90, 90	90, 103.445 (1), 90	90, 105.820 (1), 90
*V* (Å^3^)	4861.56 (19)	2217.40 (10)	1514.00 (5)	1510.54 (5)
*Z*	8	4	4	4
Radiation type	Mo *K*α	Mo *K*α	Mo *K*α	Mo *K*α
μ (mm^−1^)	0.33	0.36	0.28	0.28
Crystal size (mm)	0.36 × 0.19 × 0.05	0.49 × 0.22 × 0.17	0.43 × 0.36 × 0.16	0.34 × 0.34 × 0.09

Data collection				
Diffractometer	Bruker D8 Venture Photon CCD area detector	Bruker D8 Venture Photon CCD area detector	Bruker D8 Venture Photon CCD area detector	Bruker D8 Venture Photon CCD area detector
Absorption correction	Integration (*XPREP*; Bruker, 2016 ▸)	Integration (*XPREP*; Bruker, 2016 ▸)	Integration (*XPREP*; Bruker, 2016 ▸)	Integration (*XPREP*; Bruker, 2016 ▸)
*T*_min_, *T*_max_	0.927, 0.986	0.887, 0.954	0.914, 0.978	0.921, 0.981
No. of measured, independent and observed [*I* > 2σ(*I*)] reflections	16830, 5858, 4131	20018, 2749, 2269	19814, 3644, 3487	26282, 3650, 3027
*R*_int_	0.047	0.037	0.047	0.052

Refinement				
*R*[*F* ^2^ > 2σ(*F* ^2^)], *wR*(*F* ^2^), *S*	0.039, 0.093, 0.95	0.031, 0.084, 1.06	0.026, 0.066, 1.07	0.039, 0.109, 1.05
No. of reflections	5858	2749	3644	3650
No. of parameters	354	173	212	212
No. of restraints	0	0	2	0
H-atom treatment	H atoms treated by a mixture of independent and constrained refinement	H atoms treated by a mixture of independent and constrained refinement	H atoms treated by a mixture of independent and constrained refinement	H atoms treated by a mixture of independent and constrained refinement
Δρ_max_, Δρ_min_ (e Å^−3^)	0.29, −0.34	0.27, −0.23	0.15, −0.16	0.55, −0.32
Absolute structure	–	–	Flack *x* determined using 1663 quotients [(*I* ^+^)-(*I* ^-^)]/[(*I* ^+^)+(*I* ^-^)] (Parsons *et al.*, 2013 ▸)	–
Absolute structure parameter	–	–	−0.010 (19)	–

Computer programs: *APEX3*, *SAINT-Plus* and *XPREP* (Bruker 2016 ▸), *SHELXS97* (Sheldrick, 2015 ▸), *SHELXL2017/1* (Sheldrick, 2015 ▸), *ORTEPIII for Windows* and *WinGX* publication routines (Farrugia, 2012 ▸) and *DIAMOND* (Brandenburg & Berndt, 1999 ▸).

## supplementary crystallographic information

### Ammonium hexamethylenetetraminium bis(2-chloro-4-nitro-benzoate) (I) Crystal data

**Table:**

H_4_N^+^·C_6_H_13_N_4_^+^·2C_7_H_3_ClNO_4_^−^	*F*(000) = 2320
*M**_r_* = 560.35	*D*_x_ = 1.531 Mg m^−^^3^
Monoclinic, *C*2/*c*	Mo *K*α radiation, λ = 0.71073 Å
Hall symbol: -C 2yc	Cell parameters from 4303 reflections
*a* = 33.6032 (8) Å	θ = 2.4–26.5°
*b* = 6.0235 (1) Å	µ = 0.33 mm^−^^1^
*c* = 28.0229 (7) Å	*T* = 173 K
β = 121.007 (1)°	Plate, yellow
*V* = 4861.56 (19) Å^3^	0.36 × 0.19 × 0.05 mm
*Z* = 8	

### Ammonium hexamethylenetetraminium bis(2-chloro-4-nitro-benzoate) (I) Data collection

**Table:**

Bruker D8 Venture Photon CCD area detector diffractometer	4131 reflections with *I* > 2σ(*I*)
Graphite monochromator	*R*_int_ = 0.047
ω scans	θ_max_ = 28.0°, θ_min_ = 1.4°
Absorption correction: integration (XPREP; Bruker, 2016)	*h* = −44→42
*T*_min_ = 0.927, *T*_max_ = 0.986	*k* = −7→7
16830 measured reflections	*l* = −34→37
5858 independent reflections	

### Ammonium hexamethylenetetraminium bis(2-chloro-4-nitro-benzoate) (I) Refinement

**Table:**

Refinement on *F*^2^	0 constraints
Least-squares matrix: full	Hydrogen site location: mixed
*R*[*F*^2^ > 2σ(*F*^2^)] = 0.039	H atoms treated by a mixture of independent and constrained refinement
*wR*(*F*^2^) = 0.093	*w* = 1/[σ^2^(*F*_o_^2^) + (0.0425*P*)^2^] where *P* = (*F*_o_^2^ + 2*F*_c_^2^)/3
*S* = 0.95	(Δ/σ)_max_ < 0.001
5858 reflections	Δρ_max_ = 0.29 e Å^−^^3^
354 parameters	Δρ_min_ = −0.34 e Å^−^^3^
0 restraints	

### Ammonium hexamethylenetetraminium bis(2-chloro-4-nitro-benzoate) (I) Special details

**Table:**

Experimental. Numerical integration absorption corrections based on indexed crystal faces were applied using the XPREP routine (Bruker, 2007)
Geometry. All esds (except the esd in the dihedral angle between two l.s. planes) are estimated using the full covariance matrix. The cell esds are taken into account individually in the estimation of esds in distances, angles and torsion angles; correlations between esds in cell parameters are only used when they are defined by crystal symmetry. An approximate (isotropic) treatment of cell esds is used for estimating esds involving l.s. planes.

### Ammonium hexamethylenetetraminium bis(2-chloro-4-nitro-benzoate) (I) Fractional atomic coordinates and isotropic or equivalent isotropic displacement parameters (Å^2^)

**Table:**

	*x*	*y*	*z*	*U*_iso_*/*U*_eq_	
C1	0.12646 (7)	0.8962 (3)	0.25907 (8)	0.0436 (5)	
H1B	0.124511	0.734282	0.263614	0.052*	
H1C	0.15024	0.92274	0.248896	0.052*	
C2	0.14211 (6)	1.2486 (3)	0.30330 (8)	0.0376 (4)	
H2B	0.152064	1.326005	0.338888	0.045*	
H2C	0.165633	1.27754	0.292835	0.045*	
C3	0.08364 (7)	1.2276 (3)	0.20814 (7)	0.0322 (4)	
H3B	0.106877	1.25849	0.197318	0.039*	
H3C	0.053299	1.285973	0.178539	0.039*	
C4	0.06273 (6)	1.2897 (3)	0.27554 (7)	0.0340 (4)	
H4B	0.032214	1.348363	0.246406	0.041*	
H4C	0.071612	1.367283	0.310805	0.041*	
C5	0.10396 (8)	0.9702 (4)	0.32528 (8)	0.0538 (6)	
H5A	0.101801	0.808914	0.330395	0.065*	
H5B	0.113292	1.044666	0.361035	0.065*	
C6	0.04479 (7)	0.9401 (3)	0.23067 (8)	0.0417 (5)	
H6A	0.013988	0.994563	0.201219	0.05*	
H6B	0.042317	0.778576	0.235157	0.05*	
N1	0.08009 (5)	0.9820 (2)	0.21368 (6)	0.0298 (3)	
N2	0.13960 (6)	1.0090 (3)	0.31095 (6)	0.0436 (4)	
N3	0.09712 (5)	1.3374 (2)	0.26001 (6)	0.0293 (3)	
N4	0.05840 (6)	1.0525 (3)	0.28254 (6)	0.0400 (4)	
H1	0.0698 (7)	0.909 (3)	0.1795 (9)	0.055 (6)*	
C7	0.06854 (5)	0.5904 (3)	0.05877 (6)	0.0239 (3)	
C8	0.08353 (5)	0.3865 (3)	0.05022 (6)	0.0242 (3)	
C9	0.07300 (5)	0.3194 (3)	−0.00250 (6)	0.0254 (4)	
H9	0.082512	0.178213	−0.008152	0.03*	
C10	0.04844 (5)	0.4631 (3)	−0.04620 (6)	0.0272 (4)	
C11	0.03282 (6)	0.6669 (3)	−0.04007 (7)	0.0304 (4)	
H11	0.015947	0.76348	−0.07092	0.037*	
C12	0.04261 (5)	0.7252 (3)	0.01254 (7)	0.0283 (4)	
H12	0.031212	0.86257	0.017342	0.034*	
C13	0.08027 (6)	0.6776 (3)	0.11575 (7)	0.0263 (4)	
N5	0.03893 (5)	0.3921 (3)	−0.10143 (6)	0.0344 (4)	
O1	0.04796 (4)	0.7787 (2)	0.11648 (5)	0.0392 (3)	
O2	0.12006 (4)	0.6506 (2)	0.15595 (5)	0.0360 (3)	
O3	0.02132 (5)	0.5272 (3)	−0.13945 (5)	0.0491 (4)	
O4	0.04925 (4)	0.2028 (2)	−0.10660 (5)	0.0442 (4)	
Cl1	0.11490 (2)	0.19844 (7)	0.10370 (2)	0.03492 (12)	
C14	0.22494 (5)	0.1024 (3)	0.09588 (6)	0.0241 (3)	
C15	0.18721 (5)	0.0359 (3)	0.04462 (6)	0.0232 (3)	
C16	0.17243 (6)	0.1593 (3)	−0.00290 (7)	0.0258 (4)	
H16	0.146226	0.11506	−0.037342	0.031*	
C17	0.19690 (6)	0.3500 (3)	0.00099 (6)	0.0268 (4)	
C18	0.23531 (6)	0.4187 (3)	0.05009 (7)	0.0296 (4)	
H18	0.252105	0.547679	0.051372	0.035*	
C19	0.24862 (6)	0.2941 (3)	0.09738 (7)	0.0278 (4)	
H19	0.274601	0.340546	0.131781	0.033*	
C20	0.24019 (5)	−0.0227 (3)	0.14930 (7)	0.0279 (4)	
N6	0.17928 (6)	0.4903 (3)	−0.04867 (6)	0.0377 (4)	
O5	0.24220 (5)	0.0883 (2)	0.18852 (5)	0.0463 (4)	
O6	0.24993 (4)	−0.22222 (19)	0.15110 (5)	0.0353 (3)	
O7	0.20330 (6)	0.6416 (3)	−0.04802 (6)	0.0652 (5)	
O8	0.14019 (5)	0.4528 (3)	−0.08803 (5)	0.0594 (4)	
Cl2	0.15515 (2)	−0.19840 (7)	0.03935 (2)	0.03327 (12)	
N1A	0.21195 (6)	0.5093 (3)	0.19658 (7)	0.0301 (3)	
H1A	0.1800 (8)	0.543 (3)	0.1788 (8)	0.046 (6)*	
H2A	0.2175 (7)	0.365 (4)	0.1897 (8)	0.045 (6)*	
H4A	0.2269 (7)	0.617 (4)	0.1824 (8)	0.056 (6)*	
H3A	0.2251 (7)	0.522 (3)	0.2341 (9)	0.046 (6)*	

### Ammonium hexamethylenetetraminium bis(2-chloro-4-nitro-benzoate) (I) Atomic displacement parameters (Å^2^)

**Table:**

	*U*^11^	*U*^22^	*U*^33^	*U*^12^	*U*^13^	*U*^23^
C1	0.0376 (10)	0.0339 (10)	0.0426 (11)	0.0118 (9)	0.0088 (9)	−0.0071 (9)
C2	0.0293 (9)	0.0417 (11)	0.0359 (10)	−0.0049 (8)	0.0125 (8)	−0.0159 (8)
C3	0.0407 (10)	0.0352 (10)	0.0257 (8)	0.0047 (8)	0.0207 (8)	0.0023 (7)
C4	0.0335 (9)	0.0428 (11)	0.0288 (9)	0.0015 (8)	0.0183 (8)	−0.0080 (8)
C5	0.0782 (16)	0.0458 (13)	0.0310 (10)	−0.0021 (12)	0.0235 (11)	0.0110 (9)
C6	0.0423 (11)	0.0425 (11)	0.0428 (11)	−0.0186 (9)	0.0237 (9)	−0.0145 (9)
N1	0.0286 (7)	0.0305 (8)	0.0267 (7)	−0.0013 (6)	0.0117 (6)	−0.0103 (6)
N2	0.0404 (9)	0.0420 (10)	0.0291 (8)	0.0126 (8)	0.0040 (7)	−0.0011 (7)
N3	0.0364 (8)	0.0255 (7)	0.0299 (7)	−0.0010 (6)	0.0198 (7)	−0.0040 (6)
N4	0.0465 (9)	0.0495 (10)	0.0296 (8)	−0.0157 (8)	0.0237 (8)	−0.0066 (7)
C7	0.0206 (7)	0.0273 (9)	0.0279 (8)	−0.0059 (7)	0.0153 (7)	−0.0069 (7)
C8	0.0239 (8)	0.0257 (8)	0.0243 (8)	−0.0042 (7)	0.0135 (7)	−0.0017 (7)
C9	0.0245 (8)	0.0282 (9)	0.0266 (8)	−0.0042 (7)	0.0155 (7)	−0.0075 (7)
C10	0.0206 (8)	0.0387 (10)	0.0217 (8)	−0.0050 (7)	0.0106 (7)	−0.0063 (7)
C11	0.0234 (8)	0.0356 (10)	0.0270 (8)	−0.0008 (7)	0.0092 (7)	0.0013 (7)
C12	0.0228 (8)	0.0278 (9)	0.0334 (9)	−0.0001 (7)	0.0139 (7)	−0.0046 (7)
C13	0.0302 (9)	0.0237 (9)	0.0310 (9)	−0.0076 (7)	0.0201 (8)	−0.0086 (7)
N5	0.0237 (7)	0.0551 (10)	0.0224 (7)	−0.0012 (7)	0.0105 (6)	−0.0038 (7)
O1	0.0282 (6)	0.0513 (8)	0.0410 (7)	−0.0063 (6)	0.0198 (6)	−0.0237 (6)
O2	0.0354 (7)	0.0425 (7)	0.0260 (6)	0.0049 (6)	0.0129 (6)	−0.0074 (5)
O3	0.0473 (8)	0.0709 (10)	0.0260 (7)	0.0071 (7)	0.0166 (6)	0.0067 (7)
O4	0.0400 (8)	0.0593 (9)	0.0312 (7)	0.0072 (7)	0.0170 (6)	−0.0145 (6)
Cl1	0.0519 (3)	0.0274 (2)	0.0269 (2)	0.0028 (2)	0.0213 (2)	0.00014 (17)
C14	0.0230 (8)	0.0225 (8)	0.0256 (8)	0.0026 (7)	0.0117 (7)	−0.0020 (7)
C15	0.0234 (8)	0.0197 (8)	0.0294 (8)	−0.0030 (6)	0.0157 (7)	−0.0046 (7)
C16	0.0247 (8)	0.0294 (9)	0.0229 (8)	−0.0031 (7)	0.0119 (7)	−0.0062 (7)
C17	0.0310 (9)	0.0280 (9)	0.0232 (8)	−0.0024 (7)	0.0153 (7)	−0.0009 (7)
C18	0.0317 (9)	0.0272 (9)	0.0319 (9)	−0.0083 (7)	0.0179 (8)	−0.0023 (7)
C19	0.0230 (8)	0.0277 (9)	0.0254 (8)	−0.0040 (7)	0.0071 (7)	−0.0035 (7)
C20	0.0233 (8)	0.0273 (9)	0.0280 (9)	0.0004 (7)	0.0096 (7)	0.0024 (7)
N6	0.0480 (10)	0.0392 (9)	0.0260 (8)	−0.0087 (8)	0.0191 (7)	−0.0007 (7)
O5	0.0737 (10)	0.0317 (7)	0.0230 (6)	0.0127 (7)	0.0174 (7)	0.0018 (6)
O6	0.0391 (7)	0.0249 (6)	0.0444 (7)	0.0065 (5)	0.0233 (6)	0.0058 (6)
O7	0.0738 (11)	0.0669 (10)	0.0432 (8)	−0.0364 (9)	0.0217 (8)	0.0112 (8)
O8	0.0607 (10)	0.0618 (10)	0.0288 (7)	−0.0197 (8)	0.0039 (7)	0.0090 (7)
Cl2	0.0287 (2)	0.0269 (2)	0.0405 (2)	−0.00875 (17)	0.01514 (19)	−0.00310 (18)
N1A	0.0353 (9)	0.0268 (8)	0.0231 (8)	−0.0002 (7)	0.0114 (7)	−0.0008 (7)

### Ammonium hexamethylenetetraminium bis(2-chloro-4-nitro-benzoate) (I) Geometric parameters (Å, º)

**Table:**

C1—N2	1.452 (2)	C9—H9	0.95
C1—N1	1.507 (2)	C10—C11	1.380 (2)
C1—H1B	0.99	C10—N5	1.473 (2)
C1—H1C	0.99	C11—C12	1.380 (2)
C2—N3	1.467 (2)	C11—H11	0.95
C2—N2	1.468 (2)	C12—H12	0.95
C2—H2B	0.99	C13—O2	1.237 (2)
C2—H2C	0.99	C13—O1	1.254 (2)
C3—N3	1.441 (2)	N5—O4	1.222 (2)
C3—N1	1.498 (2)	N5—O3	1.2243 (19)
C3—H3B	0.99	C14—C19	1.391 (2)
C3—H3C	0.99	C14—C15	1.398 (2)
C4—N3	1.458 (2)	C14—C20	1.510 (2)
C4—N4	1.460 (2)	C15—C16	1.375 (2)
C4—H4B	0.99	C15—Cl2	1.7349 (16)
C4—H4C	0.99	C16—C17	1.384 (2)
C5—N4	1.461 (3)	C16—H16	0.95
C5—N2	1.465 (3)	C17—C18	1.379 (2)
C5—H5A	0.99	C17—N6	1.467 (2)
C5—H5B	0.99	C18—C19	1.382 (2)
C6—N4	1.448 (2)	C18—H18	0.95
C6—N1	1.510 (2)	C19—H19	0.95
C6—H6A	0.99	C20—O6	1.2399 (19)
C6—H6B	0.99	C20—O5	1.258 (2)
N1—H1	0.94 (2)	N6—O7	1.2113 (19)
C7—C12	1.391 (2)	N6—O8	1.2247 (19)
C7—C8	1.394 (2)	N1A—H1A	0.94 (2)
C7—C13	1.527 (2)	N1A—H2A	0.93 (2)
C8—C9	1.390 (2)	N1A—H4A	1.02 (2)
C8—Cl1	1.7357 (16)	N1A—H3A	0.91 (2)
C9—C10	1.374 (2)		
			
N2—C1—N1	109.47 (14)	C8—C7—C13	124.12 (15)
N2—C1—H1B	109.8	C9—C8—C7	121.47 (15)
N1—C1—H1B	109.8	C9—C8—Cl1	115.88 (13)
N2—C1—H1C	109.8	C7—C8—Cl1	122.62 (12)
N1—C1—H1C	109.8	C10—C9—C8	118.11 (15)
H1B—C1—H1C	108.2	C10—C9—H9	120.9
N3—C2—N2	111.53 (14)	C8—C9—H9	120.9
N3—C2—H2B	109.3	C9—C10—C11	122.78 (15)
N2—C2—H2B	109.3	C9—C10—N5	117.32 (15)
N3—C2—H2C	109.3	C11—C10—N5	119.90 (15)
N2—C2—H2C	109.3	C12—C11—C10	117.57 (16)
H2B—C2—H2C	108	C12—C11—H11	121.2
N3—C3—N1	110.42 (14)	C10—C11—H11	121.2
N3—C3—H3B	109.6	C11—C12—C7	122.45 (16)
N1—C3—H3B	109.6	C11—C12—H12	118.8
N3—C3—H3C	109.6	C7—C12—H12	118.8
N1—C3—H3C	109.6	O2—C13—O1	125.97 (15)
H3B—C3—H3C	108.1	O2—C13—C7	118.86 (15)
N3—C4—N4	112.46 (14)	O1—C13—C7	115.12 (15)
N3—C4—H4B	109.1	O4—N5—O3	123.84 (15)
N4—C4—H4B	109.1	O4—N5—C10	118.31 (15)
N3—C4—H4C	109.1	O3—N5—C10	117.85 (16)
N4—C4—H4C	109.1	C19—C14—C15	118.09 (15)
H4B—C4—H4C	107.8	C19—C14—C20	119.18 (14)
N4—C5—N2	112.37 (15)	C15—C14—C20	122.72 (14)
N4—C5—H5A	109.1	C16—C15—C14	121.59 (15)
N2—C5—H5A	109.1	C16—C15—Cl2	117.30 (12)
N4—C5—H5B	109.1	C14—C15—Cl2	121.01 (13)
N2—C5—H5B	109.1	C15—C16—C17	117.97 (15)
H5A—C5—H5B	107.9	C15—C16—H16	121
N4—C6—N1	110.20 (14)	C17—C16—H16	121
N4—C6—H6A	109.6	C18—C17—C16	122.72 (15)
N1—C6—H6A	109.6	C18—C17—N6	119.13 (15)
N4—C6—H6B	109.6	C16—C17—N6	118.04 (14)
N1—C6—H6B	109.6	C17—C18—C19	117.93 (15)
H6A—C6—H6B	108.1	C17—C18—H18	121
C3—N1—C1	108.86 (14)	C19—C18—H18	121
C3—N1—C6	108.38 (14)	C18—C19—C14	121.64 (15)
C1—N1—C6	108.37 (15)	C18—C19—H19	119.2
C3—N1—H1	111.0 (13)	C14—C19—H19	119.2
C1—N1—H1	112.1 (13)	O6—C20—O5	125.82 (16)
C6—N1—H1	108.0 (13)	O6—C20—C14	118.14 (15)
C1—N2—C5	109.25 (17)	O5—C20—C14	116.04 (14)
C1—N2—C2	108.94 (16)	O7—N6—O8	123.31 (16)
C5—N2—C2	108.27 (16)	O7—N6—C17	118.61 (15)
C3—N3—C4	108.97 (14)	O8—N6—C17	118.03 (15)
C3—N3—C2	108.73 (14)	H1A—N1A—H2A	112.6 (17)
C4—N3—C2	108.61 (14)	H1A—N1A—H4A	108.4 (17)
C6—N4—C4	108.95 (14)	H2A—N1A—H4A	108.8 (17)
C6—N4—C5	108.66 (16)	H1A—N1A—H3A	109.6 (17)
C4—N4—C5	107.91 (15)	H2A—N1A—H3A	106.8 (17)
C12—C7—C8	117.56 (15)	H4A—N1A—H3A	110.7 (17)
C12—C7—C13	118.28 (14)		
			
N3—C3—N1—C1	−58.93 (19)	N5—C10—C11—C12	179.91 (15)
N3—C3—N1—C6	58.74 (18)	C10—C11—C12—C7	2.2 (2)
N2—C1—N1—C3	58.6 (2)	C8—C7—C12—C11	−2.1 (2)
N2—C1—N1—C6	−59.1 (2)	C13—C7—C12—C11	175.80 (15)
N4—C6—N1—C3	−58.4 (2)	C12—C7—C13—O2	−136.62 (16)
N4—C6—N1—C1	59.64 (19)	C8—C7—C13—O2	41.1 (2)
N1—C1—N2—C5	58.9 (2)	C12—C7—C13—O1	41.0 (2)
N1—C1—N2—C2	−59.2 (2)	C8—C7—C13—O1	−141.29 (16)
N4—C5—N2—C1	−60.0 (2)	C9—C10—N5—O4	6.9 (2)
N4—C5—N2—C2	58.6 (2)	C11—C10—N5—O4	−173.10 (15)
N3—C2—N2—C1	60.8 (2)	C9—C10—N5—O3	−172.66 (15)
N3—C2—N2—C5	−57.87 (19)	C11—C10—N5—O3	7.3 (2)
N1—C3—N3—C4	−59.01 (18)	C19—C14—C15—C16	−2.5 (2)
N1—C3—N3—C2	59.21 (19)	C20—C14—C15—C16	176.37 (15)
N4—C4—N3—C3	59.83 (18)	C19—C14—C15—Cl2	−178.82 (12)
N4—C4—N3—C2	−58.46 (18)	C20—C14—C15—Cl2	0.0 (2)
N2—C2—N3—C3	−60.51 (19)	C14—C15—C16—C17	1.8 (2)
N2—C2—N3—C4	57.93 (19)	Cl2—C15—C16—C17	178.27 (12)
N1—C6—N4—C4	58.2 (2)	C15—C16—C17—C18	0.6 (3)
N1—C6—N4—C5	−59.1 (2)	C15—C16—C17—N6	−175.74 (15)
N3—C4—N4—C6	−59.57 (19)	C16—C17—C18—C19	−2.1 (3)
N3—C4—N4—C5	58.23 (19)	N6—C17—C18—C19	174.17 (16)
N2—C5—N4—C6	59.7 (2)	C17—C18—C19—C14	1.4 (3)
N2—C5—N4—C4	−58.3 (2)	C15—C14—C19—C18	0.8 (2)
C12—C7—C8—C9	−0.1 (2)	C20—C14—C19—C18	−178.03 (16)
C13—C7—C8—C9	−177.82 (15)	C19—C14—C20—O6	−124.75 (17)
C12—C7—C8—Cl1	−178.35 (12)	C15—C14—C20—O6	56.4 (2)
C13—C7—C8—Cl1	3.9 (2)	C19—C14—C20—O5	54.6 (2)
C7—C8—C9—C10	2.0 (2)	C15—C14—C20—O5	−124.17 (18)
Cl1—C8—C9—C10	−179.62 (12)	C18—C17—N6—O7	12.8 (3)
C8—C9—C10—C11	−1.9 (2)	C16—C17—N6—O7	−170.81 (17)
C8—C9—C10—N5	178.06 (14)	C18—C17—N6—O8	−164.69 (17)
C9—C10—C11—C12	−0.1 (2)	C16—C17—N6—O8	11.7 (2)

### Ammonium hexamethylenetetraminium bis(2-chloro-4-nitro-benzoate) (I) Hydrogen-bond geometry (Å, º)

**Table:**

*D*—H···*A*	*D*—H	H···*A*	*D*···*A*	*D*—H···*A*
N1—H1···O1	0.94 (2)	1.71 (2)	2.6564 (18)	177 (2)
N1*A*—H1*A*···O2	0.94 (2)	1.89 (2)	2.817 (2)	168 (2)
N1*A*—H2*A*···O5	0.93 (2)	1.87 (2)	2.784 (2)	167 (2)
N1*A*—H3*A*···O5^i^	0.91 (2)	1.90 (2)	2.803 (2)	171 (2)
N1*A*—H4*A*···O6^ii^	1.02 (2)	1.73 (2)	2.747 (2)	173 (2)

Symmetry codes: (i) −*x*+1/2, *y*+1/2, −*z*+1/2; (ii) *x*, *y*+1, *z*.

### Hexamethylenetetraminium hydrogen bis(2-chloro-4-nitro-benzoate) (II) Crystal data

**Table:**

C_6_H_13_N_4_^+^·C_14_H_7_Cl_2_N_2_O_8_^−^	*F*(000) = 1120
*M**_r_* = 543.32	*D*_x_ = 1.627 Mg m^−^^3^
Orthorhombic, *P**n**m**a*	Mo *K*α radiation, λ = 0.71073 Å
Hall symbol: -P 2ac 2n	Cell parameters from 7712 reflections
*a* = 8.2777 (2) Å	θ = 2.9–28.3°
*b* = 19.7942 (5) Å	µ = 0.36 mm^−^^1^
*c* = 13.5331 (4) Å	*T* = 173 K
*V* = 2217.40 (10) Å^3^	Block, yellow
*Z* = 4	0.49 × 0.22 × 0.17 mm

### Hexamethylenetetraminium hydrogen bis(2-chloro-4-nitro-benzoate) (II) Data collection

**Table:**

Bruker D8 Venture Photon CCD area detector diffractometer	2269 reflections with *I* > 2σ(*I*)
Graphite monochromator	*R*_int_ = 0.037
ω scans	θ_max_ = 28.0°, θ_min_ = 1.8°
Absorption correction: integration (XPREP; Bruker, 2016)	*h* = −9→10
*T*_min_ = 0.887, *T*_max_ = 0.954	*k* = −26→26
20018 measured reflections	*l* = −15→17
2749 independent reflections	

### Hexamethylenetetraminium hydrogen bis(2-chloro-4-nitro-benzoate) (II) Refinement

**Table:**

Refinement on *F*^2^	0 constraints
Least-squares matrix: full	Hydrogen site location: mixed
*R*[*F*^2^ > 2σ(*F*^2^)] = 0.031	H atoms treated by a mixture of independent and constrained refinement
*wR*(*F*^2^) = 0.084	*w* = 1/[σ^2^(*F*_o_^2^) + (0.0459*P*)^2^ + 0.3817*P*] where *P* = (*F*_o_^2^ + 2*F*_c_^2^)/3
*S* = 1.06	(Δ/σ)_max_ = 0.001
2749 reflections	Δρ_max_ = 0.27 e Å^−^^3^
173 parameters	Δρ_min_ = −0.23 e Å^−^^3^
0 restraints	

### Hexamethylenetetraminium hydrogen bis(2-chloro-4-nitro-benzoate) (II) Special details

**Table:**

Experimental. Numerical integration absorption corrections based on indexed crystal faces were applied using the XPREP routine (Bruker, 2016)
Geometry. All esds (except the esd in the dihedral angle between two l.s. planes) are estimated using the full covariance matrix. The cell esds are taken into account individually in the estimation of esds in distances, angles and torsion angles; correlations between esds in cell parameters are only used when they are defined by crystal symmetry. An approximate (isotropic) treatment of cell esds is used for estimating esds involving l.s. planes.

### Hexamethylenetetraminium hydrogen bis(2-chloro-4-nitro-benzoate) (II) Fractional atomic coordinates and isotropic or equivalent isotropic displacement parameters (Å^2^)

**Table:**

	*x*	*y*	*z*	*U*_iso_*/*U*_eq_	Occ. (<1)
C1	0.5362 (2)	0.25	0.60139 (15)	0.0293 (4)	
H1A	0.602402	0.209478	0.615413	0.035*	0.5
H1B	0.602403	0.290522	0.615413	0.035*	0.5
C2	0.29732 (18)	0.31010 (6)	0.64113 (11)	0.0316 (3)	
H2A	0.201625	0.3107	0.684998	0.038*	
H2B	0.361744	0.351156	0.655045	0.038*	
C3	0.38433 (16)	0.31232 (7)	0.47447 (11)	0.0302 (3)	
H3A	0.449124	0.353372	0.487692	0.036*	
H3B	0.350359	0.313181	0.404342	0.036*	
C4	0.1490 (2)	0.25	0.51788 (16)	0.0317 (4)	
H4A	0.051092	0.249999	0.559915	0.038*	
H4B	0.113777	0.25	0.447969	0.038*	
N1	0.48529 (18)	0.25	0.49400 (12)	0.0266 (3)	
N2	0.3952 (2)	0.25	0.66325 (12)	0.0298 (3)	
N3	0.24353 (13)	0.31154 (5)	0.53769 (9)	0.0284 (2)	
H1	0.575 (3)	0.25	0.4540 (19)	0.050 (7)*	
C5	0.91417 (14)	0.41404 (6)	0.36290 (9)	0.0225 (2)	
C6	0.84727 (14)	0.47610 (6)	0.38838 (9)	0.0224 (3)	
C7	0.93805 (15)	0.53490 (6)	0.38801 (9)	0.0236 (3)	
H7	0.890704	0.577243	0.403921	0.028*	
C8	1.09962 (15)	0.52991 (6)	0.36376 (9)	0.0240 (3)	
C9	1.17365 (15)	0.46922 (6)	0.34177 (9)	0.0259 (3)	
H9	1.286207	0.46701	0.328452	0.031*	
C10	1.07902 (15)	0.41200 (6)	0.33976 (9)	0.0254 (3)	
H10	1.126815	0.370043	0.322226	0.03*	
C11	0.82257 (15)	0.34800 (6)	0.36070 (10)	0.0263 (3)	
N4	1.19475 (14)	0.59237 (5)	0.35791 (8)	0.0290 (2)	
O1	0.85862 (14)	0.31125 (5)	0.28588 (7)	0.0377 (3)	
H2	0.83408	0.270874	0.297686	0.057*	0.5
O2	0.72967 (12)	0.33353 (5)	0.42674 (8)	0.0413 (3)	
O3	1.33995 (11)	0.58735 (5)	0.34060 (9)	0.0404 (3)	
O4	1.12464 (12)	0.64612 (5)	0.36731 (9)	0.0407 (3)	
Cl1	0.64344 (4)	0.48455 (2)	0.41656 (3)	0.03169 (11)	

### Hexamethylenetetraminium hydrogen bis(2-chloro-4-nitro-benzoate) (II) Atomic displacement parameters (Å^2^)

**Table:**

	*U*^11^	*U*^22^	*U*^33^	*U*^12^	*U*^13^	*U*^23^
C1	0.0282 (9)	0.0237 (8)	0.0360 (10)	0	−0.0040 (8)	0
C2	0.0363 (7)	0.0243 (6)	0.0343 (7)	0.0015 (5)	0.0073 (6)	−0.0047 (5)
C3	0.0284 (7)	0.0277 (6)	0.0344 (7)	0.0016 (5)	0.0039 (5)	0.0090 (5)
C4	0.0231 (9)	0.0317 (9)	0.0404 (11)	0	0.0011 (8)	0
N1	0.0220 (7)	0.0262 (7)	0.0316 (8)	0	0.0065 (6)	0
N2	0.0383 (9)	0.0237 (7)	0.0276 (8)	0	0.0015 (7)	0
N3	0.0249 (5)	0.0251 (5)	0.0351 (6)	0.0026 (4)	0.0046 (5)	0.0028 (4)
C5	0.0238 (6)	0.0242 (6)	0.0194 (5)	0.0018 (5)	0.0011 (5)	0.0031 (5)
C6	0.0199 (6)	0.0278 (6)	0.0196 (6)	0.0041 (5)	0.0017 (4)	0.0025 (5)
C7	0.0265 (6)	0.0238 (6)	0.0207 (6)	0.0049 (5)	−0.0003 (5)	0.0013 (5)
C8	0.0261 (6)	0.0253 (6)	0.0207 (6)	−0.0015 (5)	0.0003 (5)	0.0021 (5)
C9	0.0216 (6)	0.0316 (6)	0.0244 (6)	0.0032 (5)	0.0035 (5)	0.0023 (5)
C10	0.0261 (6)	0.0237 (6)	0.0262 (6)	0.0065 (5)	0.0041 (5)	0.0015 (5)
C11	0.0265 (6)	0.0243 (6)	0.0281 (7)	0.0030 (5)	−0.0003 (5)	0.0030 (5)
N4	0.0302 (6)	0.0292 (6)	0.0277 (6)	−0.0029 (5)	0.0015 (5)	0.0026 (4)
O1	0.0618 (7)	0.0215 (5)	0.0299 (5)	−0.0019 (4)	0.0082 (5)	0.0000 (4)
O2	0.0399 (6)	0.0337 (5)	0.0503 (7)	−0.0100 (5)	0.0201 (5)	−0.0041 (5)
O3	0.0273 (5)	0.0398 (6)	0.0541 (7)	−0.0066 (4)	0.0075 (4)	0.0006 (5)
O4	0.0406 (6)	0.0248 (5)	0.0566 (7)	−0.0009 (4)	0.0045 (5)	−0.0009 (5)
Cl1	0.02016 (16)	0.03606 (19)	0.0388 (2)	0.00424 (12)	0.00556 (12)	−0.00421 (14)

### Hexamethylenetetraminium hydrogen bis(2-chloro-4-nitro-benzoate) (II) Geometric parameters (Å, º)

**Table:**

C1—N2	1.436 (2)	C5—C6	1.3909 (17)
C1—N1	1.513 (2)	C5—C10	1.4006 (17)
C1—H1A	0.99	C5—C11	1.5115 (17)
C1—H1B	0.99	C6—C7	1.3854 (17)
C2—N3	1.4693 (19)	C6—Cl1	1.7378 (12)
C2—N2	1.4703 (16)	C7—C8	1.3806 (17)
C2—H2A	0.99	C7—H7	0.95
C2—H2B	0.99	C8—C9	1.3811 (17)
C3—N3	1.4460 (16)	C8—N4	1.4679 (16)
C3—N1	1.5132 (15)	C9—C10	1.3775 (18)
C3—H3A	0.99	C9—H9	0.95
C3—H3B	0.99	C10—H10	0.95
C4—N3^i^	1.4725 (14)	C11—O2	1.2133 (16)
C4—N3	1.4725 (14)	C11—O1	1.2820 (16)
C4—H4A	0.99	N4—O4	1.2185 (14)
C4—H4B	0.99	N4—O3	1.2286 (14)
N1—H1	0.92 (3)	O1—H2	0.84
			
N2—C1—N1	109.51 (14)	C1—N2—C2^i^	109.21 (10)
N2—C1—H1A	109.8	C2—N2—C2^i^	108.03 (15)
N1—C1—H1A	109.8	C3—N3—C2	108.64 (11)
N2—C1—H1B	109.8	C3—N3—C4	109.24 (12)
N1—C1—H1B	109.8	C2—N3—C4	108.57 (12)
H1A—C1—H1B	108.2	C6—C5—C10	117.96 (11)
N3—C2—N2	112.13 (11)	C6—C5—C11	124.68 (10)
N3—C2—H2A	109.2	C10—C5—C11	117.34 (11)
N2—C2—H2A	109.2	C7—C6—C5	121.67 (11)
N3—C2—H2B	109.2	C7—C6—Cl1	116.53 (9)
N2—C2—H2B	109.2	C5—C6—Cl1	121.73 (9)
H2A—C2—H2B	107.9	C8—C7—C6	117.79 (11)
N3—C3—N1	109.46 (11)	C8—C7—H7	121.1
N3—C3—H3A	109.8	C6—C7—H7	121.1
N1—C3—H3A	109.8	C7—C8—C9	122.90 (12)
N3—C3—H3B	109.8	C7—C8—N4	118.18 (11)
N1—C3—H3B	109.8	C9—C8—N4	118.88 (11)
H3A—C3—H3B	108.2	C10—C9—C8	117.86 (11)
N3^i^—C4—N3	111.64 (14)	C10—C9—H9	121.1
N3^i^—C4—H4A	109.3	C8—C9—H9	121.1
N3—C4—H4A	109.3	C9—C10—C5	121.73 (11)
N3^i^—C4—H4B	109.3	C9—C10—H10	119.1
N3—C4—H4B	109.3	C5—C10—H10	119.1
H4A—C4—H4B	108	O2—C11—O1	126.54 (12)
C1—N1—C3	108.75 (10)	O2—C11—C5	120.51 (12)
C1—N1—C3^i^	108.75 (10)	O1—C11—C5	112.92 (11)
C3—N1—C3^i^	109.21 (14)	O4—N4—O3	123.81 (11)
C1—N1—H1	110.0 (16)	O4—N4—C8	118.31 (10)
C3—N1—H1	110.1 (8)	O3—N4—C8	117.83 (11)
C3^i^—N1—H1	110.1 (8)	C11—O1—H2	109.5
C1—N2—C2	109.21 (10)		
			
N2—C1—N1—C3	59.41 (9)	C5—C6—C7—C8	−1.59 (18)
N2—C1—N1—C3^i^	−59.42 (9)	Cl1—C6—C7—C8	−178.61 (9)
N3—C3—N1—C1	−59.65 (14)	C6—C7—C8—C9	−1.09 (19)
N3—C3—N1—C3^i^	58.89 (18)	C6—C7—C8—N4	176.73 (11)
N1—C1—N2—C2	−58.97 (10)	C7—C8—C9—C10	3.08 (19)
N1—C1—N2—C2^i^	58.97 (10)	N4—C8—C9—C10	−174.73 (11)
N3—C2—N2—C1	60.31 (15)	C8—C9—C10—C5	−2.47 (19)
N3—C2—N2—C2^i^	−58.36 (18)	C6—C5—C10—C9	−0.04 (19)
N1—C3—N3—C2	59.30 (14)	C11—C5—C10—C9	−178.59 (11)
N1—C3—N3—C4	−58.98 (15)	C6—C5—C11—O2	−43.34 (19)
N2—C2—N3—C3	−60.24 (14)	C10—C5—C11—O2	135.11 (14)
N2—C2—N3—C4	58.46 (15)	C6—C5—C11—O1	138.58 (13)
N3^i^—C4—N3—C3	60.40 (19)	C10—C5—C11—O1	−42.97 (16)
N3^i^—C4—N3—C2	−57.92 (18)	C7—C8—N4—O4	−5.45 (18)
C10—C5—C6—C7	2.13 (18)	C9—C8—N4—O4	172.47 (12)
C11—C5—C6—C7	−179.43 (11)	C7—C8—N4—O3	176.84 (12)
C10—C5—C6—Cl1	179.00 (9)	C9—C8—N4—O3	−5.24 (18)
C11—C5—C6—Cl1	−2.56 (18)		

Symmetry code: (i) *x*, −*y*+1/2, *z*.

### Hexamethylenetetraminium hydrogen bis(2-chloro-4-nitro-benzoate) (II) Hydrogen-bond geometry (Å, º)

**Table:**

*D*—H···*A*	*D*—H	H···*A*	*D*···*A*	*D*—H···*A*
N1—H1···O2	0.92 (3)	2.12 (2)	2.7667 (15)	126 (1)

### Hexamethylenetetraminium 2-chloro-4-nitro-benzoate (IIIa) Crystal data

**Table:**

C_6_H_13_N_4_^+^·C_7_H_3_ClNO_4_^−^	*F*(000) = 712
*M**_r_* = 341.76	*D*_x_ = 1.499 Mg m^−^^3^
Monoclinic, *C**c*	Mo *K*α radiation, λ = 0.71073 Å
Hall symbol: C -2yc	Cell parameters from 8081 reflections
*a* = 5.9049 (1) Å	θ = 2.6–28.2°
*b* = 21.9330 (4) Å	µ = 0.28 mm^−^^1^
*c* = 12.0194 (2) Å	*T* = 173 K
β = 103.445 (1)°	Block, yellow
*V* = 1514.00 (5) Å^3^	0.43 × 0.36 × 0.16 mm
*Z* = 4	

### Hexamethylenetetraminium 2-chloro-4-nitro-benzoate (IIIa) Data collection

**Table:**

Bruker D8 Venture Photon CCD area detector diffractometer	3487 reflections with *I* > 2σ(*I*)
Graphite monochromator	*R*_int_ = 0.047
ω scans	θ_max_ = 28.0°, θ_min_ = 1.9°
Absorption correction: integration (XPREP; Bruker, 2016)	*h* = −7→7
*T*_min_ = 0.914, *T*_max_ = 0.978	*k* = −28→28
19814 measured reflections	*l* = −15→15
3644 independent reflections	

### Hexamethylenetetraminium 2-chloro-4-nitro-benzoate (IIIa) Refinement

**Table:**

Refinement on *F*^2^	Hydrogen site location: mixed
Least-squares matrix: full	H atoms treated by a mixture of independent and constrained refinement
*R*[*F*^2^ > 2σ(*F*^2^)] = 0.026	*w* = 1/[σ^2^(*F*_o_^2^) + (0.0432*P*)^2^] where *P* = (*F*_o_^2^ + 2*F*_c_^2^)/3
*wR*(*F*^2^) = 0.066	(Δ/σ)_max_ = 0.001
*S* = 1.07	Δρ_max_ = 0.15 e Å^−^^3^
3644 reflections	Δρ_min_ = −0.16 e Å^−^^3^
212 parameters	Absolute structure: Flack *x* determined using 1663 quotients [(*I*^+^)-(*I*^-^)]/[(*I*^+^)+(*I*^-^)] (Parsons *et al.*, 2013)
2 restraints	Absolute structure parameter: −0.010 (19)
0 constraints	

### Hexamethylenetetraminium 2-chloro-4-nitro-benzoate (IIIa) Special details

**Table:**

Experimental. Numerical integration absorption corrections based on indexed crystal faces were applied using the XPREP routine (Bruker, 2007)
Geometry. All esds (except the esd in the dihedral angle between two l.s. planes) are estimated using the full covariance matrix. The cell esds are taken into account individually in the estimation of esds in distances, angles and torsion angles; correlations between esds in cell parameters are only used when they are defined by crystal symmetry. An approximate (isotropic) treatment of cell esds is used for estimating esds involving l.s. planes.

### Hexamethylenetetraminium 2-chloro-4-nitro-benzoate (IIIa) Fractional atomic coordinates and isotropic or equivalent isotropic displacement parameters (Å^2^)

**Table:**

	*x*	*y*	*z*	*U*_iso_*/*U*_eq_	
C1	1.0080 (3)	0.72838 (9)	0.47292 (17)	0.0302 (4)	
H1A	1.096685	0.737124	0.551861	0.036*	
H1B	1.049816	0.686893	0.452137	0.036*	
C2	0.9342 (4)	0.75914 (10)	0.27795 (17)	0.0319 (4)	
H2A	0.976526	0.788891	0.224231	0.038*	
H2B	0.976087	0.717955	0.25558	0.038*	
C3	0.6207 (3)	0.71808 (8)	0.34676 (16)	0.0286 (4)	
H3A	0.658855	0.676431	0.325098	0.034*	
H3B	0.451188	0.719959	0.341854	0.034*	
C4	0.6238 (4)	0.82334 (9)	0.3026 (2)	0.0350 (4)	
H4A	0.661827	0.853586	0.248535	0.042*	
H4B	0.454371	0.825696	0.297832	0.042*	
C5	1.0013 (4)	0.83387 (9)	0.4256 (2)	0.0391 (5)	
H5A	1.088446	0.843625	0.504334	0.047*	
H5B	1.045272	0.864199	0.3733	0.047*	
C6	0.6902 (4)	0.79500 (9)	0.49725 (19)	0.0343 (4)	
H6A	0.521113	0.797405	0.493412	0.041*	
H6B	0.774748	0.804796	0.576425	0.041*	
N1	0.7517 (3)	0.73145 (7)	0.46727 (14)	0.0260 (3)	
N2	1.0680 (3)	0.77256 (8)	0.39466 (16)	0.0320 (4)	
N3	0.6820 (3)	0.76185 (7)	0.26875 (14)	0.0291 (3)	
N4	0.7511 (3)	0.83861 (7)	0.41911 (17)	0.0347 (4)	
H1	0.701 (5)	0.7025 (12)	0.521 (3)	0.047 (7)*	
C7	0.4837 (3)	0.55022 (8)	0.59523 (16)	0.0247 (4)	
C8	0.5755 (3)	0.51846 (8)	0.69629 (15)	0.0235 (3)	
C9	0.4580 (3)	0.47118 (8)	0.73393 (16)	0.0242 (3)	
H9	0.523206	0.449697	0.802544	0.029*	
C10	0.2400 (3)	0.45652 (8)	0.66667 (17)	0.0252 (4)	
C11	0.1378 (3)	0.48766 (9)	0.56796 (17)	0.0317 (4)	
H11	−0.014186	0.477454	0.525556	0.038*	
C12	0.2629 (4)	0.53414 (9)	0.53253 (18)	0.0330 (4)	
H12	0.196649	0.555527	0.463953	0.04*	
C13	0.6155 (3)	0.60098 (8)	0.55257 (15)	0.0258 (4)	
N5	0.1104 (3)	0.40717 (7)	0.70493 (14)	0.0282 (3)	
O1	0.5964 (3)	0.65311 (6)	0.59380 (14)	0.0443 (4)	
O2	0.7254 (2)	0.58844 (6)	0.48014 (12)	0.0323 (3)	
O3	−0.0851 (3)	0.39514 (8)	0.64851 (16)	0.0452 (4)	
O4	0.2033 (3)	0.37964 (7)	0.79287 (13)	0.0393 (4)	
Cl1	0.84686 (7)	0.53900 (2)	0.77974 (4)	0.03201 (12)	

### Hexamethylenetetraminium 2-chloro-4-nitro-benzoate (IIIa) Atomic displacement parameters (Å^2^)

**Table:**

	*U*^11^	*U*^22^	*U*^33^	*U*^12^	*U*^13^	*U*^23^
C1	0.0260 (10)	0.0383 (10)	0.0253 (9)	0.0055 (8)	0.0038 (7)	0.0053 (8)
C2	0.0363 (11)	0.0347 (10)	0.0284 (10)	0.0076 (8)	0.0149 (9)	0.0089 (8)
C3	0.0262 (9)	0.0276 (9)	0.0304 (10)	−0.0036 (7)	0.0035 (7)	−0.0036 (7)
C4	0.0320 (11)	0.0278 (9)	0.0429 (11)	0.0092 (8)	0.0043 (9)	0.0053 (8)
C5	0.0335 (11)	0.0321 (10)	0.0508 (13)	−0.0118 (8)	0.0079 (10)	−0.0032 (9)
C6	0.0374 (11)	0.0320 (10)	0.0378 (11)	0.0008 (8)	0.0176 (9)	−0.0103 (8)
N1	0.0294 (8)	0.0256 (7)	0.0248 (8)	−0.0012 (6)	0.0100 (6)	−0.0006 (6)
N2	0.0205 (8)	0.0382 (8)	0.0382 (9)	0.0007 (7)	0.0087 (7)	0.0060 (7)
N3	0.0308 (9)	0.0275 (8)	0.0268 (8)	0.0046 (6)	0.0019 (7)	0.0034 (6)
N4	0.0359 (9)	0.0230 (7)	0.0457 (10)	−0.0005 (6)	0.0109 (8)	−0.0045 (7)
C7	0.0301 (10)	0.0238 (8)	0.0201 (8)	0.0006 (7)	0.0056 (7)	−0.0003 (6)
C8	0.0224 (8)	0.0240 (7)	0.0225 (8)	−0.0004 (7)	0.0015 (7)	−0.0022 (6)
C9	0.0260 (9)	0.0231 (8)	0.0220 (8)	−0.0001 (6)	0.0024 (7)	0.0014 (6)
C10	0.0271 (9)	0.0227 (8)	0.0250 (9)	−0.0025 (6)	0.0044 (7)	−0.0014 (6)
C11	0.0287 (10)	0.0369 (10)	0.0252 (9)	−0.0044 (8)	−0.0023 (8)	0.0008 (8)
C12	0.0360 (11)	0.0365 (10)	0.0225 (9)	−0.0033 (8)	−0.0012 (8)	0.0061 (7)
C13	0.0310 (9)	0.0267 (8)	0.0191 (8)	−0.0010 (7)	0.0046 (7)	0.0019 (6)
N5	0.0291 (8)	0.0235 (7)	0.0310 (8)	−0.0044 (6)	0.0048 (7)	−0.0034 (6)
O1	0.0744 (12)	0.0273 (7)	0.0427 (9)	−0.0096 (7)	0.0370 (9)	−0.0058 (6)
O2	0.0353 (8)	0.0317 (7)	0.0331 (7)	0.0026 (5)	0.0147 (6)	0.0024 (6)
O3	0.0341 (8)	0.0444 (8)	0.0503 (9)	−0.0169 (7)	−0.0039 (7)	0.0037 (7)
O4	0.0414 (9)	0.0321 (7)	0.0410 (9)	−0.0084 (6)	0.0027 (7)	0.0114 (6)
Cl1	0.0265 (2)	0.0358 (2)	0.0297 (2)	−0.00838 (18)	−0.00158 (16)	0.00365 (19)

### Hexamethylenetetraminium 2-chloro-4-nitro-benzoate (IIIa) Geometric parameters (Å, º)

**Table:**

C1—N2	1.450 (3)	C6—N1	1.505 (2)
C1—N1	1.501 (3)	C6—H6A	0.99
C1—H1A	0.99	C6—H6B	0.99
C1—H1B	0.99	N1—H1	1.00 (3)
C2—N3	1.469 (3)	C7—C12	1.392 (3)
C2—N2	1.471 (3)	C7—C8	1.396 (3)
C2—H2A	0.99	C7—C13	1.514 (2)
C2—H2B	0.99	C8—C9	1.381 (3)
C3—N3	1.445 (2)	C8—Cl1	1.7405 (18)
C3—N1	1.504 (2)	C9—C10	1.390 (3)
C3—H3A	0.99	C9—H9	0.95
C3—H3B	0.99	C10—C11	1.380 (3)
C4—N4	1.466 (3)	C10—N5	1.460 (2)
C4—N3	1.472 (3)	C11—C12	1.383 (3)
C4—H4A	0.99	C11—H11	0.95
C4—H4B	0.99	C12—H12	0.95
C5—N4	1.466 (3)	C13—O2	1.232 (2)
C5—N2	1.473 (3)	C13—O1	1.262 (2)
C5—H5A	0.99	N5—O3	1.224 (2)
C5—H5B	0.99	N5—O4	1.230 (2)
C6—N4	1.443 (3)		
			
N2—C1—N1	109.65 (15)	C3—N1—C6	108.21 (15)
N2—C1—H1A	109.7	C1—N1—H1	113.2 (17)
N1—C1—H1A	109.7	C3—N1—H1	109.4 (17)
N2—C1—H1B	109.7	C6—N1—H1	107.9 (15)
N1—C1—H1B	109.7	C1—N2—C2	109.08 (16)
H1A—C1—H1B	108.2	C1—N2—C5	109.07 (17)
N3—C2—N2	111.95 (15)	C2—N2—C5	107.93 (17)
N3—C2—H2A	109.2	C3—N3—C2	109.06 (15)
N2—C2—H2A	109.2	C3—N3—C4	108.64 (15)
N3—C2—H2B	109.2	C2—N3—C4	108.30 (17)
N2—C2—H2B	109.2	C6—N4—C5	108.62 (17)
H2A—C2—H2B	107.9	C6—N4—C4	108.69 (16)
N3—C3—N1	110.18 (15)	C5—N4—C4	108.72 (18)
N3—C3—H3A	109.6	C12—C7—C8	118.04 (17)
N1—C3—H3A	109.6	C12—C7—C13	119.58 (16)
N3—C3—H3B	109.6	C8—C7—C13	122.38 (17)
N1—C3—H3B	109.6	C9—C8—C7	122.48 (17)
H3A—C3—H3B	108.1	C9—C8—Cl1	118.08 (14)
N4—C4—N3	111.86 (15)	C7—C8—Cl1	119.43 (14)
N4—C4—H4A	109.2	C8—C9—C10	116.82 (17)
N3—C4—H4A	109.2	C8—C9—H9	121.6
N4—C4—H4B	109.2	C10—C9—H9	121.6
N3—C4—H4B	109.2	C11—C10—C9	123.11 (17)
H4A—C4—H4B	107.9	C11—C10—N5	118.76 (17)
N4—C5—N2	112.12 (16)	C9—C10—N5	118.09 (16)
N4—C5—H5A	109.2	C10—C11—C12	118.13 (18)
N2—C5—H5A	109.2	C10—C11—H11	120.9
N4—C5—H5B	109.2	C12—C11—H11	120.9
N2—C5—H5B	109.2	C11—C12—C7	121.37 (18)
H5A—C5—H5B	107.9	C11—C12—H12	119.3
N4—C6—N1	110.32 (15)	C7—C12—H12	119.3
N4—C6—H6A	109.6	O2—C13—O1	126.16 (17)
N1—C6—H6A	109.6	O2—C13—C7	118.14 (16)
N4—C6—H6B	109.6	O1—C13—C7	115.67 (16)
N1—C6—H6B	109.6	O3—N5—O4	123.01 (17)
H6A—C6—H6B	108.1	O3—N5—C10	118.76 (16)
C1—N1—C3	108.81 (15)	O4—N5—C10	118.23 (15)
C1—N1—C6	109.14 (15)		
			
N2—C1—N1—C3	59.36 (19)	N3—C4—N4—C5	57.9 (2)
N2—C1—N1—C6	−58.5 (2)	C12—C7—C8—C9	1.7 (3)
N3—C3—N1—C1	−59.28 (19)	C13—C7—C8—C9	−178.40 (17)
N3—C3—N1—C6	59.19 (19)	C12—C7—C8—Cl1	−177.75 (15)
N4—C6—N1—C1	59.0 (2)	C13—C7—C8—Cl1	2.2 (2)
N4—C6—N1—C3	−59.2 (2)	C7—C8—C9—C10	−0.6 (3)
N1—C1—N2—C2	−59.2 (2)	Cl1—C8—C9—C10	178.85 (14)
N1—C1—N2—C5	58.5 (2)	C8—C9—C10—C11	−1.5 (3)
N3—C2—N2—C1	59.7 (2)	C8—C9—C10—N5	−179.32 (16)
N3—C2—N2—C5	−58.7 (2)	C9—C10—C11—C12	2.5 (3)
N4—C5—N2—C1	−60.1 (2)	N5—C10—C11—C12	−179.77 (18)
N4—C5—N2—C2	58.3 (2)	C10—C11—C12—C7	−1.3 (3)
N1—C3—N3—C2	58.6 (2)	C8—C7—C12—C11	−0.7 (3)
N1—C3—N3—C4	−59.30 (19)	C13—C7—C12—C11	179.37 (18)
N2—C2—N3—C3	−59.3 (2)	C12—C7—C13—O2	−83.2 (2)
N2—C2—N3—C4	58.8 (2)	C8—C7—C13—O2	96.9 (2)
N4—C4—N3—C3	60.2 (2)	C12—C7—C13—O1	95.1 (2)
N4—C4—N3—C2	−58.1 (2)	C8—C7—C13—O1	−84.8 (2)
N1—C6—N4—C5	−58.8 (2)	C11—C10—N5—O3	−0.3 (3)
N1—C6—N4—C4	59.3 (2)	C9—C10—N5—O3	177.59 (18)
N2—C5—N4—C6	59.9 (2)	C11—C10—N5—O4	179.86 (18)
N2—C5—N4—C4	−58.2 (2)	C9—C10—N5—O4	−2.3 (3)
N3—C4—N4—C6	−60.2 (2)		

### Hexamethylenetetraminium 2-chloro-4-nitro-benzoate (IIIa) Hydrogen-bond geometry (Å, º)

**Table:**

*D*—H···*A*	*D*—H	H···*A*	*D*···*A*	*D*—H···*A*
N1—H1···O1	1.00 (3)	1.60 (3)	2.599 (2)	173 (3)

### Hexamethylenetetraminium 2-chloro-4-nitro-benzoate (IIIb) Crystal data

**Table:**

C_6_H_13_N_4_^+^·C_7_H_3_ClNO_4_^−^	*F*(000) = 712
*M**_r_* = 341.76	*D*_x_ = 1.503 Mg m^−^^3^
Monoclinic, *P*2_1_/*c*	Mo *K*α radiation, λ = 0.71073 Å
Hall symbol: -P 2ybc	Cell parameters from 8173 reflections
*a* = 12.0663 (2) Å	θ = 2.7–28.3°
*b* = 19.5741 (4) Å	µ = 0.28 mm^−^^1^
*c* = 6.6473 (1) Å	*T* = 173 K
β = 105.820 (1)°	Flint, yellow
*V* = 1510.54 (5) Å^3^	0.34 × 0.34 × 0.09 mm
*Z* = 4	

### Hexamethylenetetraminium 2-chloro-4-nitro-benzoate (IIIb) Data collection

**Table:**

Bruker D8 Venture Photon CCD area detector diffractometer	3027 reflections with *I* > 2σ(*I*)
Graphite monochromator	*R*_int_ = 0.052
ω scans	θ_max_ = 28°, θ_min_ = 1.8°
Absorption correction: integration (XPREP; Bruker, 2016)	*h* = −15→15
*T*_min_ = 0.921, *T*_max_ = 0.981	*k* = −25→25
26282 measured reflections	*l* = −7→8
3650 independent reflections	

### Hexamethylenetetraminium 2-chloro-4-nitro-benzoate (IIIb) Refinement

**Table:**

Refinement on *F*^2^	0 constraints
Least-squares matrix: full	Hydrogen site location: mixed
*R*[*F*^2^ > 2σ(*F*^2^)] = 0.039	H atoms treated by a mixture of independent and constrained refinement
*wR*(*F*^2^) = 0.109	*w* = 1/[σ^2^(*F*_o_^2^) + (0.054*P*)^2^ + 0.5922*P*] where *P* = (*F*_o_^2^ + 2*F*_c_^2^)/3
*S* = 1.05	(Δ/σ)_max_ = 0.001
3650 reflections	Δρ_max_ = 0.55 e Å^−^^3^
212 parameters	Δρ_min_ = −0.32 e Å^−^^3^
0 restraints	

### Hexamethylenetetraminium 2-chloro-4-nitro-benzoate (IIIb) Special details

**Table:**

Experimental. Numerical integration absorption corrections based on indexed crystal faces were applied using the XPREP routine (Bruker, 2016)
Geometry. All esds (except the esd in the dihedral angle between two l.s. planes) are estimated using the full covariance matrix. The cell esds are taken into account individually in the estimation of esds in distances, angles and torsion angles; correlations between esds in cell parameters are only used when they are defined by crystal symmetry. An approximate (isotropic) treatment of cell esds is used for estimating esds involving l.s. planes.

### Hexamethylenetetraminium 2-chloro-4-nitro-benzoate (IIIb) Fractional atomic coordinates and isotropic or equivalent isotropic displacement parameters (Å^2^)

**Table:**

	*x*	*y*	*z*	*U*_iso_*/*U*_eq_	
C1	0.47109 (13)	0.06841 (8)	0.8298 (2)	0.0271 (3)	
H1A	0.476631	0.018936	0.803213	0.033*	
H1B	0.546088	0.08373	0.921587	0.033*	
C2	0.37372 (14)	0.15425 (8)	0.9693 (2)	0.0276 (3)	
H2A	0.313223	0.162548	1.041406	0.033*	
H2B	0.447916	0.170366	1.062001	0.033*	
C3	0.43573 (13)	0.18246 (7)	0.6683 (2)	0.0244 (3)	
H3A	0.510444	0.199335	0.757087	0.029*	
H3B	0.417076	0.208139	0.534971	0.029*	
C4	0.23625 (12)	0.16842 (8)	0.6381 (2)	0.0266 (3)	
H4A	0.17462	0.176786	0.707533	0.032*	
H4B	0.216747	0.19411	0.50475	0.032*	
C5	0.27060 (14)	0.05744 (8)	0.7927 (3)	0.0286 (3)	
H5A	0.274979	0.008016	0.764242	0.034*	
H5B	0.208987	0.064229	0.863228	0.034*	
C6	0.32952 (12)	0.08285 (8)	0.4889 (2)	0.0243 (3)	
H6A	0.310535	0.107689	0.354087	0.029*	
H6B	0.333422	0.033463	0.459442	0.029*	
N1	0.44482 (11)	0.10695 (6)	0.6253 (2)	0.0224 (3)	
N2	0.38141 (12)	0.08021 (6)	0.9327 (2)	0.0272 (3)	
N3	0.34674 (10)	0.19364 (6)	0.77319 (19)	0.0236 (3)	
N4	0.24105 (10)	0.09499 (6)	0.5939 (2)	0.0253 (3)	
H1	0.5012 (17)	0.0994 (10)	0.563 (3)	0.034 (5)*	
C7	0.75647 (12)	0.14049 (7)	0.3243 (2)	0.0245 (3)	
C8	0.86231 (13)	0.10840 (7)	0.4116 (2)	0.0250 (3)	
C9	0.95324 (12)	0.11511 (8)	0.3214 (3)	0.0283 (3)	
H9	1.025178	0.093435	0.380783	0.034*	
C10	0.93535 (13)	0.15426 (8)	0.1431 (2)	0.0291 (3)	
C11	0.83262 (14)	0.18678 (8)	0.0513 (3)	0.0312 (3)	
H11	0.822882	0.213174	−0.072295	0.037*	
C12	0.74455 (13)	0.17962 (8)	0.1452 (3)	0.0295 (3)	
H12	0.673439	0.202156	0.085609	0.035*	
C13	0.65228 (13)	0.13753 (8)	0.4116 (3)	0.0276 (3)	
N5	1.03144 (12)	0.16391 (9)	0.0488 (2)	0.0407 (4)	
O1	0.61912 (11)	0.08068 (6)	0.4561 (2)	0.0409 (3)	
O2	0.60096 (11)	0.19263 (6)	0.4134 (2)	0.0434 (3)	
O3	1.02236 (14)	0.20679 (8)	−0.0877 (3)	0.0594 (4)	
O4	1.11599 (12)	0.12771 (11)	0.1120 (2)	0.0706 (6)	
Cl1	0.88646 (4)	0.06116 (2)	0.64016 (6)	0.03601 (13)	

### Hexamethylenetetraminium 2-chloro-4-nitro-benzoate (IIIb) Atomic displacement parameters (Å^2^)

**Table:**

	*U*^11^	*U*^22^	*U*^33^	*U*^12^	*U*^13^	*U*^23^
C1	0.0267 (7)	0.0216 (7)	0.0312 (8)	0.0035 (6)	0.0047 (6)	0.0047 (6)
C2	0.0346 (8)	0.0249 (7)	0.0237 (7)	−0.0002 (6)	0.0088 (6)	−0.0012 (6)
C3	0.0265 (7)	0.0156 (6)	0.0333 (8)	−0.0003 (5)	0.0116 (6)	0.0010 (6)
C4	0.0223 (7)	0.0268 (7)	0.0310 (8)	0.0051 (6)	0.0076 (6)	−0.0002 (6)
C5	0.0312 (8)	0.0241 (7)	0.0346 (8)	−0.0056 (6)	0.0160 (6)	−0.0001 (6)
C6	0.0247 (7)	0.0239 (7)	0.0249 (7)	−0.0001 (6)	0.0077 (6)	−0.0021 (6)
N1	0.0216 (6)	0.0179 (6)	0.0298 (6)	0.0015 (4)	0.0110 (5)	0.0020 (5)
N2	0.0350 (7)	0.0222 (6)	0.0247 (6)	0.0002 (5)	0.0090 (5)	0.0032 (5)
N3	0.0265 (6)	0.0191 (6)	0.0269 (6)	0.0014 (5)	0.0103 (5)	0.0004 (5)
N4	0.0209 (6)	0.0260 (6)	0.0292 (6)	−0.0003 (5)	0.0072 (5)	−0.0023 (5)
C7	0.0198 (7)	0.0216 (7)	0.0329 (8)	−0.0033 (5)	0.0089 (6)	−0.0067 (6)
C8	0.0231 (7)	0.0239 (7)	0.0273 (7)	−0.0025 (6)	0.0058 (6)	−0.0041 (6)
C9	0.0181 (7)	0.0333 (8)	0.0325 (8)	0.0000 (6)	0.0054 (6)	−0.0070 (6)
C10	0.0225 (7)	0.0356 (8)	0.0317 (8)	−0.0050 (6)	0.0115 (6)	−0.0082 (7)
C11	0.0305 (8)	0.0309 (8)	0.0334 (8)	−0.0023 (6)	0.0103 (7)	0.0014 (7)
C12	0.0233 (7)	0.0280 (8)	0.0368 (8)	0.0020 (6)	0.0076 (6)	−0.0010 (6)
C13	0.0219 (7)	0.0274 (8)	0.0359 (8)	−0.0027 (6)	0.0117 (6)	−0.0060 (6)
N5	0.0271 (7)	0.0621 (10)	0.0355 (8)	−0.0076 (7)	0.0129 (6)	−0.0112 (7)
O1	0.0368 (7)	0.0242 (6)	0.0723 (9)	−0.0010 (5)	0.0331 (6)	−0.0005 (6)
O2	0.0391 (7)	0.0293 (6)	0.0702 (9)	0.0047 (5)	0.0291 (7)	0.0018 (6)
O3	0.0622 (9)	0.0590 (9)	0.0723 (11)	−0.0087 (7)	0.0443 (8)	0.0089 (8)
O4	0.0281 (7)	0.1466 (18)	0.0404 (8)	0.0219 (9)	0.0149 (6)	0.0116 (9)
Cl1	0.0355 (2)	0.0380 (2)	0.0323 (2)	−0.00006 (17)	0.00555 (16)	0.00463 (16)

### Hexamethylenetetraminium 2-chloro-4-nitro-benzoate (IIIb) Geometric parameters (Å, º)

**Table:**

C1—N2	1.448 (2)	C6—N1	1.5140 (19)
C1—N1	1.5105 (19)	C6—H6A	0.99
C1—H1A	0.99	C6—H6B	0.99
C1—H1B	0.99	N1—H1	0.90 (2)
C2—N3	1.4724 (19)	C7—C12	1.390 (2)
C2—N2	1.4766 (19)	C7—C8	1.399 (2)
C2—H2A	0.99	C7—C13	1.522 (2)
C2—H2B	0.99	C8—C9	1.393 (2)
C3—N3	1.4475 (18)	C8—Cl1	1.7340 (16)
C3—N1	1.5150 (18)	C9—C10	1.378 (2)
C3—H3A	0.99	C9—H9	0.95
C3—H3B	0.99	C10—C11	1.379 (2)
C4—N4	1.4714 (19)	C10—N5	1.473 (2)
C4—N3	1.4746 (19)	C11—C12	1.379 (2)
C4—H4A	0.99	C11—H11	0.95
C4—H4B	0.99	C12—H12	0.95
C5—N4	1.469 (2)	C13—O1	1.2454 (19)
C5—N2	1.474 (2)	C13—O2	1.2454 (19)
C5—H5A	0.99	N5—O3	1.219 (2)
C5—H5B	0.99	N5—O4	1.219 (2)
C6—N4	1.4454 (19)		
			
N2—C1—N1	110.18 (11)	C6—N1—C3	108.36 (11)
N2—C1—H1A	109.6	C1—N1—H1	109.4 (12)
N1—C1—H1A	109.6	C6—N1—H1	111.2 (12)
N2—C1—H1B	109.6	C3—N1—H1	110.4 (12)
N1—C1—H1B	109.6	C1—N2—C5	108.64 (12)
H1A—C1—H1B	108.1	C1—N2—C2	108.84 (12)
N3—C2—N2	112.14 (12)	C5—N2—C2	108.28 (12)
N3—C2—H2A	109.2	C3—N3—C2	109.46 (12)
N2—C2—H2A	109.2	C3—N3—C4	108.80 (12)
N3—C2—H2B	109.2	C2—N3—C4	107.99 (11)
N2—C2—H2B	109.2	C6—N4—C5	108.79 (12)
H2A—C2—H2B	107.9	C6—N4—C4	109.36 (11)
N3—C3—N1	109.86 (11)	C5—N4—C4	108.80 (12)
N3—C3—H3A	109.7	C12—C7—C8	118.18 (14)
N1—C3—H3A	109.7	C12—C7—C13	116.36 (13)
N3—C3—H3B	109.7	C8—C7—C13	125.45 (14)
N1—C3—H3B	109.7	C9—C8—C7	121.08 (14)
H3A—C3—H3B	108.2	C9—C8—Cl1	117.62 (12)
N4—C4—N3	111.73 (11)	C7—C8—Cl1	121.27 (12)
N4—C4—H4A	109.3	C10—C9—C8	117.76 (14)
N3—C4—H4A	109.3	C10—C9—H9	121.1
N4—C4—H4B	109.3	C8—C9—H9	121.1
N3—C4—H4B	109.3	C9—C10—C11	123.26 (14)
H4A—C4—H4B	107.9	C9—C10—N5	118.72 (14)
N4—C5—N2	111.88 (12)	C11—C10—N5	118.00 (15)
N4—C5—H5A	109.2	C12—C11—C10	117.61 (15)
N2—C5—H5A	109.2	C12—C11—H11	121.2
N4—C5—H5B	109.2	C10—C11—H11	121.2
N2—C5—H5B	109.2	C11—C12—C7	122.11 (14)
H5A—C5—H5B	107.9	C11—C12—H12	118.9
N4—C6—N1	109.79 (12)	C7—C12—H12	118.9
N4—C6—H6A	109.7	O1—C13—O2	125.36 (14)
N1—C6—H6A	109.7	O1—C13—C7	118.49 (13)
N4—C6—H6B	109.7	O2—C13—C7	115.79 (14)
N1—C6—H6B	109.7	O3—N5—O4	123.66 (16)
H6A—C6—H6B	108.2	O3—N5—C10	118.92 (15)
C1—N1—C6	108.45 (11)	O4—N5—C10	117.42 (16)
C1—N1—C3	109.01 (11)		
			
N2—C1—N1—C6	−58.92 (15)	N3—C4—N4—C5	58.67 (15)
N2—C1—N1—C3	58.86 (15)	C12—C7—C8—C9	−0.3 (2)
N4—C6—N1—C1	59.13 (15)	C13—C7—C8—C9	−178.99 (14)
N4—C6—N1—C3	−59.07 (14)	C12—C7—C8—Cl1	177.65 (11)
N3—C3—N1—C1	−58.31 (15)	C13—C7—C8—Cl1	−1.0 (2)
N3—C3—N1—C6	59.54 (15)	C7—C8—C9—C10	−0.2 (2)
N1—C1—N2—C5	58.93 (15)	Cl1—C8—C9—C10	−178.17 (12)
N1—C1—N2—C2	−58.77 (15)	C8—C9—C10—C11	0.1 (2)
N4—C5—N2—C1	−60.34 (15)	C8—C9—C10—N5	178.05 (14)
N4—C5—N2—C2	57.72 (16)	C9—C10—C11—C12	0.4 (2)
N3—C2—N2—C1	59.64 (16)	N5—C10—C11—C12	−177.55 (14)
N3—C2—N2—C5	−58.29 (16)	C10—C11—C12—C7	−0.9 (2)
N1—C3—N3—C2	58.23 (15)	C8—C7—C12—C11	0.9 (2)
N1—C3—N3—C4	−59.57 (15)	C13—C7—C12—C11	179.66 (14)
N2—C2—N3—C3	−59.64 (16)	C12—C7—C13—O1	131.29 (16)
N2—C2—N3—C4	58.66 (16)	C8—C7—C13—O1	−50.0 (2)
N4—C4—N3—C3	60.13 (15)	C12—C7—C13—O2	−42.2 (2)
N4—C4—N3—C2	−58.59 (15)	C8—C7—C13—O2	136.53 (17)
N1—C6—N4—C5	−59.65 (15)	C9—C10—N5—O3	−168.41 (16)
N1—C6—N4—C4	59.06 (15)	C11—C10—N5—O3	9.7 (2)
N2—C5—N4—C6	60.87 (15)	C9—C10—N5—O4	12.0 (2)
N2—C5—N4—C4	−58.19 (15)	C11—C10—N5—O4	−169.91 (17)
N3—C4—N4—C6	−60.04 (16)		

### Hexamethylenetetraminium 2-chloro-4-nitro-benzoate (IIIb) Hydrogen-bond geometry (Å, º)

**Table:**

*D*—H···*A*	*D*—H	H···*A*	*D*···*A*	*D*—H···*A*
N1—H1···O1	0.90 (2)	1.80 (2)	2.6911 (17)	175.7 (19)
